# Diagnostic ability and its influenced factors of ultrasound-guided percutaneous pleural needle biopsy diagnosis for malignant pleural mesothelioma

**DOI:** 10.3389/fsurg.2022.1022505

**Published:** 2022-09-26

**Authors:** Yuxin Zhang, Jiaxin Tang, Xinghua Zhou, Wuxi Chen, Shiyu Zhang, Yuqin Li, Dazhi Zhou, Liantu He, Qing Tang

**Affiliations:** ^1^ Department of Ultrasound, First Affiliated Hospital of Guangzhou Medical University, Guangzhou, China; ^2^ The State Key Laboratory of Respiratory Disease, Guangzhou Institute of Respiratory Disease, the First Affiliated Hospital, Guangzhou Medical University, Guangzhou, China

**Keywords:** ultrasound, pleural biopsy, malignant pleural mesothelioma, diagnostic accuracy, influenced factors

## Abstract

**Background:**

Malignant pleural mesothelioma (MPM) is a highly invasive malignant tumor. Ultrasound guidance has the advantages of real-time, convenience and nonradiative. We sought to identify diagnostic value and its influenced factors of ultrasound-guided percutaneous pleural needle biopsy (US-PPNB) for MPM.

**Methods:**

Patients who underwent US-PPNB between March 2014 and March 2020 and were finally diagnosed with MPM were retrospectively analyzed. We retrospectively analyzed the US-PPNBs pathological results of all patients clinically confirmed as MPM, and divided US-PPNBs into correctly and incorrectly diagnosed groups. Patient, thoracic, and biopsy variables that affected diagnostic accuracy were assessed. All variables significant on univariate analyses were subjected to multivariate logistic regression to identify significant predictors of diagnostic accuracy. We derived cutoffs for all significant continuous variables and used the Mantel–Haenszel test to determine whether the diagnostic accuracy of US-PPNB for MPM increased with pleural thickness.

**Results:**

In total, 49 patients with clinically confirmed MPM underwent US-PPNB; 37 diagnoses were correct and 12 were incorrect (accuracy = 75.5%). The pleura was significantly thicker in the correctly diagnosed group (*p* < 0.001). The pleural thickness cutoff was 4.15 mm and diagnostic accuracy increased with pleural thickness grade (*p* for trend <0.05). The diagnostic accuracy was significantly higher when 16-G rather than 18-G biopsy needles were used (*p* < 0.05). Multivariate logistic regression showed that pleural thickness (odds ratio: 17.2, 95% confidence interval: 2.8–104.1, *p* = 0.002) and needle size (odds ratio: 6.8, 95% confidence interval: 1.0–44.5, *p* = 0.044) independently predicted diagnostic accuracy.

**Conclusion:**

US-PPNB afforded high MPM diagnostic accuracy, and pleural thickness and needle size significantly impacted accuracy.

## Introduction

Malignant pleural mesothelioma (MPM) (both diffused and focal) is a highly invasive malignant tumor commonly caused by exposure to asbestos (1). Although MPM is rare, the global incidence has risen steadily over the past decade and the prognosis is poor (2). Early MPM is usually asymptomatic and diagnosis is therefore challenging. Patients suspected of MPM should undergo radiographic examinations, as these provide valuable information for diagnosis and staging (2–4). However, the diagnosis and tumor biological information must be confirmed/obtained *via* invasive biopsy followed by pathological examination (2, 5, 6). Pleural biopsy methods include blind closed needle biopsy, computed tomography-guided percutaneous pleural needle biopsy (CT-PPNB), ultrasound-guided percutaneous pleural needle biopsy (US-PPNB), and thoracoscopic pleural biopsy. Although the latter is considered the gold standard for MPM diagnosis, it is expensive and can be traumatic (5, 6). Hence, minimally invasive pleural biopsy, such as US-PPNB, may be more appropriate for frail patients and those lacking pleural effusion. However, blind closed needle biopsy is rarely used in most developed countries given the poor yield, and cannot be recommended as a first-line modality for investigating MPM (2). PPNB guided by computed tomography (CT) or ultrasound (US) has high diagnostic utility and is very safe. The diagnostic accuracy of image-guided PPNB for MPM was reported as 83%–88%, with CT typically used for guidance (7–9). US guidance provides real-time information, is more convenient than CT, and is not irradiative, and has thus been recommended as the preferred imaging modality for pleural effusions and guidance of minimally invasive biopsy (6). The overall diagnostic rate of US-PPNB was reported as 63%–94% (10–13). However, the diagnostic accuracy for MPM remains unclear, given the rarity of the condition and the few relevant studies.

Pleural thickness affects the diagnostic accuracy of CT-PPNB for malignant pleural diseases in general, and MPM in particular (9, 14). The sensitivity of image-guided cutting needle MPM biopsy is satisfactory when the pleural thickness is ≤5 mm (9). However, although both pleural thickness and cutting needle size affect the overall accuracy of US-guided PPNB, factors affecting the diagnostic accuracy for MPM remain unclear.

Here, we sought to identify diagnostic value and its influenced factors of US-PPNB for MPM.

## Materials and methods

### Study design

This study was approved by the Scientific Research Ethics Review Committee of the First Affiliated Hospital of Guangzhou Medical University (approval no. 2018-14). Informed consent was obtained from each patient prior to ultrasound-guided percutaneous pleural needle biopsy. Patients who underwent US-PPNB between March 2014 and March 2020 were retrospectively investigated and the patients with clinical diagnosis of MPM would be finally included and analysed in this study; all patients showed unexplained pleural thickening or effusion prior to US-PPNB.

### US-PPNB

All US-PPNBs were performed by two clinicians working together with at least 5 years of interventional experience. After the acquisitional and interpretation of chest CT scans, low- and/or high-frequency US (MyLab 90; Esaote, Genoa, Italy) was used to collect information on pleural effusion, the pleura, and blood flow. To maximize accuracy, the thickest point of the pleura or a focally thickened region was used for biopsy whenever possible. However, the thickest pleural region on CT or US was not selected if it was difficult to guarantee a safe puncture path. In such cases, a thinner but more accessible pleural region was chosen. If B-mode US could not identify a necrotic area inside a pleural lesion, or the pleura was not clearly visible, contrast-enhanced ultrasound (CEUS) was employed at the discretion of the operators. A 2.4-ml bolus of contrast agent (SonoVue; Bracco, Milan, Italy) was injected and non-enhanced areas were avoided.

The biopsy plan was decided by consensus. Operator 1 (a sonographer) assessed the pleural condition and provided real-time guidance. Operator 2 used an 18- or 16-G automated cutting needle with a specimen notch of 20 mm (MC1816 Max Core; Bard Inc., Providence, NJ, USA) to perform biopsy with patients under local anesthesia [2% (w/v) lidocaine]. The tip of the needle was inserted through a guide channel into the chest wall at least 22 mm from the lung tissue. The number of punctures depended on specimen quality and patient tolerance. Generally, 2–4 punctures were performed. However, if more tissue was required, 1–3 additional punctures were made if the patient could tolerated it.

### Pathological examination and accuracy of US-PPNB

All US-PPNB specimens were fixed in 10% (v/v) formalin and sent for histopathological examination. All samples were stained with hematoxylin and eosin (H/E) and examined by two pathologists with at least 5 years of experience. If MPM was suspected or diagnosed, further immunohistochemical tests (staining for Napsin A, CK5/6, WT1, TTF1, P40, etc.) were performed.

If the pathological diagnosis of the tissue acquired from US-PPNB was MPM, it was considered that the US-PPNB was true positive. In addition, we would conduct clinical or imaging follow-up for patients who had a negative US-PPNB (insufficient samples or benign diagnosis) at regular intervals. And all patients included in this study were followed up for at least 1 year. If patients with negative US-PPNB were still not diagnosed with MPM after 1 year follow-up, the diagnosis of MPM will be excluded. We retrospectively analyzed the US-PPNBs pathological results of all patients clinically confirmed as MPM, and divided US-PPNBs into correctly and incorrectly diagnosed groups.

### Variables and analyses

Patient, thoracic, and biopsy variables that might affect the diagnostic accuracy of US-PLNB for MPM were assessed. Patient factors included age, sex and TNM stage. Thoracic factors included pleural thickness and effusion. Pleural thickness was measured at the puncture point identified by US. Pleural effusion was divided into dry (no effusion or localized effusion <10 mm) and non-dry (effusion >10 mm) types (15). Biopsy factors included the use of contrast agent, number of punctures, needle size (18- or 16-G), and location (left or right thorax).

### Statistical analyses

All analyses were performed with SPSS software (ver. 22.0; SPSS Inc., Chicago, IL, USA). Continuous variables are expressed as means with standard deviations and categorical variables as frequencies or percentages. In univariate analyses, differences between continuous variables were examined using the independent-samples *t*-test or Mann–Whitney *U* test. We plotted receiver operator curves (ROCs) and obtained area under the curve (AUC), cutoff, sensitivity, and specificity values for all significant continuous variables. The chi-squared or Fisher exact test was used to analyze differences in categorical variables. Finally, all variables significant in univariate analyses were subjected to multivariate logistic regression to identify significant predictors of diagnostic accuracy. We used the Mantel–Haenszel trend test to determine whether diagnostic accuracy increased with pleural thickness grade. A *p*-value <0.05 was considered statistically significant.

## Results

### Demographic characteristics

A total of 49 patients with clinically confirmed MPM underwent US-PPNB from March 2014 to March 2020 [29 males and 20 females; mean age = 61.4 ± 12.1 years (range: 22–85 years)]. Among the 49 patients diagnosed with mesothelioma, 38 patients were finally diagnosed as epithelioid mesothelioma, 6 as sarcomatoid mesothelioma, 4 as biphasic mesothelioma, but one patient failed to be had a clearly subclassification. Among 49 cases, there were 11 patients in the TNM stage of I, 8 patients in the TNM stage of III, and 30 patients in the TNM stage of IV. In total, 45 patients underwent US-PPNB once, 2 US-PPNB twice, and 2 US-PPNB three times (55 US-PPNBs in total). We analyzed only the first US-PPNBs. The pathological results of the first US-PPNBs in 49 patients were negative in 12 cases and positive in 37 cases. Hence, 37 and 12 had correct and incorrect diagnoses, respectively (accuracy = 75.5%). Among the 37 cases of positive US-PPNBs, 29 patients were finally diagnosed as epithelioid mesothelioma, 5 as sarcomatoid mesothelioma, 2 as biphasic mesothelioma, but one patient failed to be had a clearly subclassification. We encountered one slight pleural reaction (chest tightness and dizzy) and one minor intrathoracic hemorrhage; both responded to symptomatic treatment. No dry MPM case developed complications. Besides, we found no mesothelioma metastases in the puncture paths during follow-up. Of the 12 cases of incorrect US-PPNB, 4 were identified *via* US-guided Abrams’ needle pleural biopsy, 3 *via* histopathological analysis after thoracoscopic biopsy, 3 *via* histopathological analysis of repeat US-PPNB tissue, 1 *via* analysis of transbronchoscopic biopsy specimens, and 1 *via* CT-PPNB (Table 1).

**Table 1:** Clinical diagnostic methods of 49 cases of MPM.

Diagnostic methods	NO.
The first time US-PPNB	37
Repeat US-PPNB	3
US-guided abrams’ needle pleural biopsy	4
Thoracoscopic biopsy	3
Transbronchoscopic biopsy	1
CT-PPNB	1
Total	49

MPM, malignant pleural mesothelioma; US, ultrasound; US-PPNB, ultrasound-guided percutaneous pleural needle biopsy; CT-PPNB, computed tomography-guided percutaneous pleural needle biopsy.

### Comparison between the two groups

**Figure 1:**
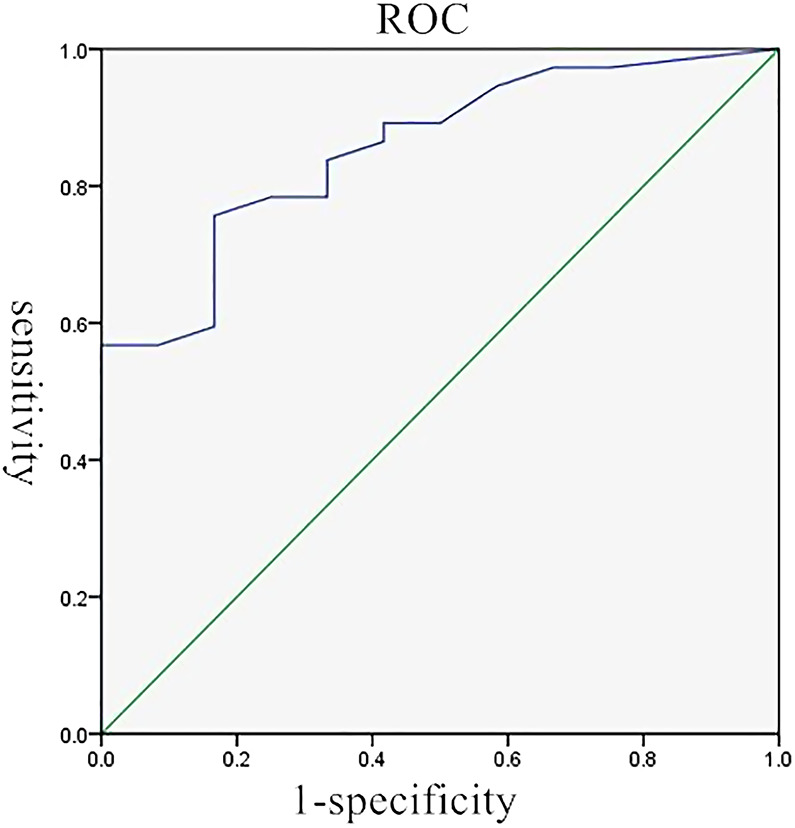
The ROC for pleural thickness in predicting the diagnostic value of US-PPNB in terms of MPM. ROC, receiver operator curve; US-PPNB, ultrasound-guided percutaneous pleural needle biopsy; MPM, malignant pleural mesothelioma.

The average pleural thicknesses of the 37 correct and 12 incorrect cases were 15.0 ± 17.0 and 3.0 ± 2.3 mm, respectively (*p* < 0.001). The pleural thickness cutoff was 4.15 mm, with a sensitivity of 75.7%, specificity of 83.3%, and AUC of 0.86 (Figure 1). Pleural thickness was further analyzed according to the cutoff of 4.15 mm (thin pleurae <4.15 mm; thick pleurae ≥4.15 mm). The correctly diagnosed group included 28 and 9 patients with thick and thin pleurae, respectively. The 12 incorrectly diagnosed patients included 10 and 2 with thick and thin pleurae, respectively. The pleural thickness differed significantly between the two groups on further analysis (*p* < 0.001) (Table 2). The diagnostic accuracy of US-PPNB for MPM increased with pleural thickness (grade 1 < 4.15; grade 2, 4.15–10; grade 3 >10 mm), as revealed by the trend test (*p* for trend <0.05).

**Table 2:** Variables between correct group and incorrect group.

	Correct group (37)	Incorrect group (12)	*p*-value
Age (year)^†^	61.0 ± 13.2	62.7 ± 8.4	0.688
Sex			0.155
Male	24	5	
Female	13	7	
TNM stage			0.427
I	7	4	
III and IV	30	8	
Pleural effusion			0.168
Dry pleura	16	2	
Non-dry pleura	21	10	
Pleural thickness (mm)^†^	15.0 ± 17.0	3.0 ± 2.3	<0.001*
Pleural thickness (cutoff value: 4.15 mm)			<0.001*
Thick pleura	28	2	
Thin pleura	9	10	
Size of cutting needle			0.043*
16G	20	2	
18G	17	10	
Use of contrast agent			0.503
Yes	14	3	
No	23	9	
Number of punctures^†^	3.6 ± 0.8	3.6 ± 1.0	0.968

^†^ Data are means ± standard deviations.

* Statistically significant (*p *< 0.05).

In total, 22 patients underwent 16-G needle biopsy (20 and 2 correctly and incorrectly diagnosed, respectively). The 27 cases who underwent 18-G biopsy needle biopsy included 17 correct and 10 incorrect diagnoses. The diagnostic accuracy of the 16-G needle was significantly higher (*p* < 0.05). We found no significant group difference in age, gender, pleural effusion status, use of contrast agent, or the number of punctures (Table 2). Multivariate logistic regression showed that pleural thickness (odds ratio 17.2, 95% confidence interval 2.8–104.1, *p* = 0.002) and needle size (odds ratio 6.8, 95% confidence interval 1.0–44.5, *p* = 0.044) independently predicted the diagnostic accuracy of US-PPNB for MPM.

## Discussion

US pleural evaluation has several advantages: the pleura lies shallowly, there is no lung gas, and pleural effusion provides contrast. Therefore, US-PPNB is recommended by several guidelines as the first-line modality for pleural biopsy (4–6). We found that the diagnostic accuracy for MPM was 75.5%. To the best of our knowledge, this is the first study to show that the size of the cutting needle and pleural thickness are significant factors in diagnostic accuracy for MPM.

In recent years, US-PPNB had become increasingly widely used. The overall diagnostic rate was reported as 63%–94%, and the sensitivity in terms of malignancy detection was 58%–85% (10–13, 16). We found that the diagnostic sensitivity was 75.5%, similar to that reported by Heilo et al. (17). Although in most cases, percutaneous cutting needle puncture is one of the preferred methods for diagnosing pleural masses and unexplained pleural effusions. However, for early stage patients with high suspicion of pleural mesothelioma, the diagnostic efficacy of thoracoscopy may be better. Because it can obtain more tumor tissue and more tumor biological information, such as fat invasion. However, most of MPM at early stage is living in seclusion. Frankly, many patients with MPM are in the late stage when they began their initial diagnosis and treatment. In this study, 78% of the patients were in the late stage of the disease (TNM stage of III and IV). These patients are no longer suitable for surgical treatment. Patients in the late stage of disease are weak and suitable for more minimally invasive diagnosis. At this time, minimally invasive methods, such as US-PPNB, should be considered. In the present study, the diagnostic accuracy of the patients in the late stage was 79% (Table 2), and all positive cases of US-PPNB met the requirements of immunohistochemical detection and subclassification. Nevertheless, it is undeniable that for patients with high suspicion of MPM, thoracoscopy should be considered when US-PPNB is negative.

**Figure 2:**
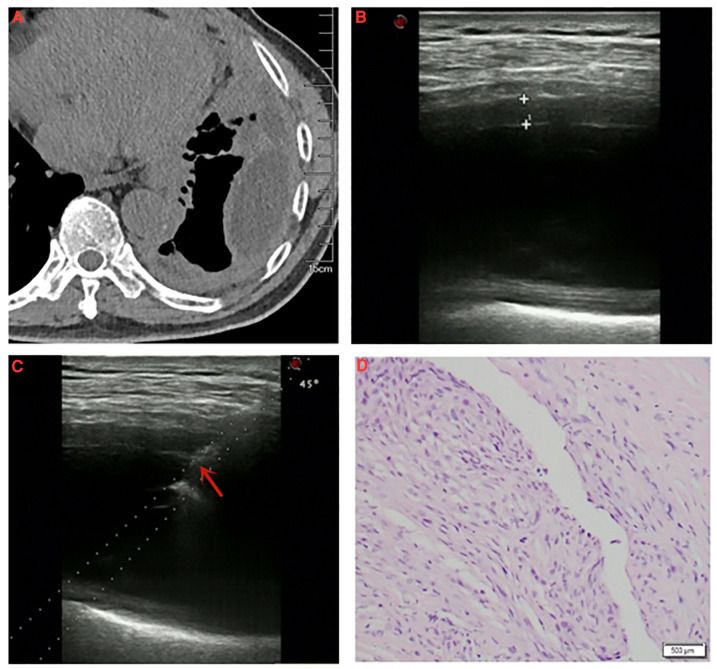
A 67-year-old male was diagnosed with pleural mesothelioma. (**A**) Chest computed tomography detected diffuse thickening of pleura with encapsulated effusion in the left thorax. (**B**) Thoracic ultrasound detected the thickness of pleura was measured to be 4.7 mm. (**C**) Specimen of thickened pleura was acquired by ultrasound-guided cutting needle biopsy (arrow: biopsy needle). (**D**) Pathological diagnosis: pleural mesothelioma (H/E, ×40).

Although US-PPNB exhibits satisfactory diagnostic accuracy, about 20% of cases are false-negatives. Variables affecting diagnostic accuracy must thus be identified. It was reported that pleural thickness significantly affected the diagnostic accuracy of US-PPNB for MPM, although only a 10-mm cutoff was used (9, 18). We also found that pleural thickness significantly affected diagnostic accuracy. The ROC curve revealed an optimal thickness cutoff of 4.15 mm. The diagnostic accuracy for thin pleurae (<4.15 mm) was 47.4%, while that for thick pleurae (≥4.15 mm) was 93.3%. We graded pleural thickness using cutoffs of 4.15 mm (this study) and 10 mm (previous studies); the diagnostic accuracy of US-PPNB for MPM increased with thickness grade (grade 1 < 4.15; grade 2 4.15–10; grade 3 >10 mm), as revealed by the trend test. Therefore, US-PPNB evaluation of patients with pleural thickness >4 mm ensures excellent diagnostic accuracy (Figure 2); accuracy improves with pleural thickness.

Cutting biopsy needles are available in a variety of sizes, and are selected according to the need to balance diagnostic accuracy with complications. We previously showed that 16-G needles were significantly better than 18-G needles in terms of overall accuracy, while the complication rates were similar (13). However, Heilo et al. found no significant difference in the diagnostic accuracy for MPM between different needles (17). Therefore, we further investigated this issue. In the early stages of adopting this technology, we gave priority to safety. Therefore, at the beginning, and when encountering frail patients, the operators tended to use 18-G needles. However, in later research, we found US-PPNB was also very safe, so 16-G needles were tended. As the accuracy afforded by the latter needle is significantly better, and as both needles are safe, 16-G needles should be preferred for MPM patients, especially those with thin pleurae.

**Figure 3:**
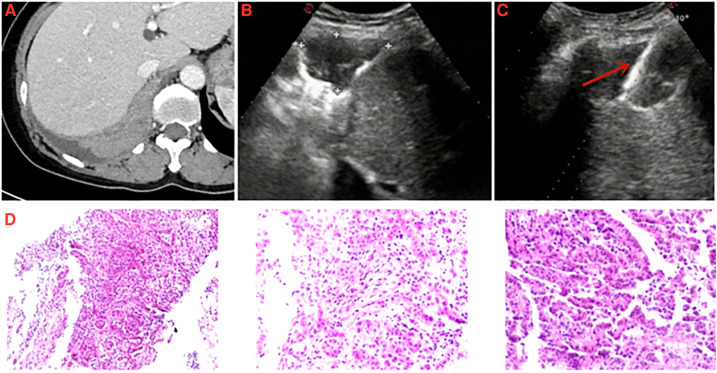
A 57-year-old female was diagnosed with pleural mesothelioma. (**A**) Chest enhanced computed tomography detected a pleural lesion in the right thorax. (**B**) Thoracic ultrasound detected the thickness of pleural lesion was measured to be 32.0 mm. (**C**) Specimen of pleural lesion was acquired by ultrasound-guided cutting needle biopsy (arrow: biopsy needle). (**D**) Pathological diagnosis: epithelioid pleural mesothelioma.

Although the results of this study showed that pleural effusion did not significantly affect the accuracy of US-PPNB to diagnose for MPM. However, it must be pointed out that pleural effusion has two sides. The pleural effusion may increase the difficulty of hemostasis. However, the pleural effusion can increase the safe distance of cutting needle biopsy and avoid to damage lung tissue. Especially when encountering biopsy of thin pleura, pleural effusion is necessary. However, dry mesothelioma is a special type of MPM. Lung tissue can be easily damaged during dry MPM PPNB given the lack of a safe path through the pleural effusion (Figure 3). It is necessary to consider the patient's breathing when adjusting the puncture angle. The real-time advantage of US can be fully revealed in this biopsy requirement. We also found that the diagnostic accuracy for “dry” MPM was 88.9% (16/18) (average pleural thickness = 24.6 mm), which is slightly higher than the rate of 80% reported by Stigt et al. (15). Besides, we previously found that the complication rate was only 6.6% after US-PPNB, and all complications were minor (13). Here, we encountered only two minor complications and no dry MPM case developed complications. Therefore, US-PPNB accurately diagnosed dry MPM with security.

This study had some limitations. First, the retrospective design may creates a risk for selection bias. Furthermore, the sample was small, and only a small number of cases underwent surgery; we were unable to compare biopsy and surgical specimens.

In conclusion, US-PPNB has high diagnostic accuracy for MPM, and the pleural thickness and size of the cutting needle significantly impact accuracy.

## Data Availability

The original contributions presented in the study are included in the article/Supplementary Material, further inquiries can be directed to the corresponding author/s
